# Characteristics of Infiltrating Immune Cells and a Predictive Immune Model for Cervical Cancer

**DOI:** 10.7150/jca.55970

**Published:** 2021-04-20

**Authors:** Ruanmin Zou, Ruihong Gu, Xia Yu, Yingying Hu, Junhui Yu, Xiangyang Xue, Xueqiong Zhu

**Affiliations:** 1 Department of Obstetrics and Gynecology, The Second Affiliated Hospital and Yuying Children's Hospital, Wenzhou Medical University, Wenzhou, People's Republic of China.; 2 Department of Microbiology and Immunology, Institute of Molecular Virology and Immunology, Institute of Tropical Medicine, College of Basic Medicine, Wenzhou Medical University, Wenzhou, People's Republic of China.; 3 Department of Obstetrics and Gynecology, The First Affiliated Hospital, Wenzhou Medical University, Wenzhou, People's Republic of China.; 4 Department of Pathology, The First Affiliated Hospital, Wenzhou Medical University, Wenzhou, People's Republic of China.

**Keywords:** cervical cancer, immune cells, prognosis, chemoradiotherapy

## Abstract

The role of infiltrating immune cells within the tumor microenvironment has received considerable attention, but their function in cervical cancer remains to be elucidated; thus, comprehensive evaluation of their predictive value is needed. Using cervical cancer samples from 406 patients, immune cell infiltration was evaluated via immunohistochemistry. CD3+, CD4+, CD8+, CD20+, CD57+, CD68+, and CD163+ cell infiltration was compared in samples from adjacent tissues and the tumor center. The associations between immune cell distributions in the tumor center, clinicopathological features, and prognosis were correlated among immune cell types. Using three immune features, an immune model was constructed based on a Cox regression analysis with the least absolute shrinkage and selection operator (lasso) penalty to derive immune risk scores.

Immune cells that infiltrated the tumor center correlated with clinicopathological characteristics and prognosis. The immune risk scores were an independent prognostic indicator and were found to predict cervical cancer prognosis as well as the effects of chemoradiotherapy. We classified patients into either high- or low-risk subgroups (namely CD4+^high^CD163+^high^CD57+^low^ and CD4+^low^CD163+^low^CD57+^high^, respectively) based on their immune scores. Significant differences were found in the 3-year overall survival of patients with high- and low-risk scores (83.0% vs. 96.6%; *P* < 0.001). High immune risk scores resulted in decreased overall survival for patients in stages IB1+IIA1, IB2+IIA2, and IIB-IV (*P* = 0.001, *P* = 0.008, and *P* = 0.044, respectively). Overall survival was significantly worse following chemoradiotherapy in high-scoring patients in stages IB1+IIA1 and IB2+IIA2 (*P* = 0.001 and *P*=0.008, respectively). Moreover, overall survival was significantly worse after radiotherapy or chemotherapy in high-scoring patients in stage IB1+IIA1 (*P* = 0.03). Our work reveals that the distribution of infiltrating immune cells affects their function in cervical cancer. Our tumor center-centric immune model effectively predicted survival, suggesting its potential use in identifying suitable candidates for chemoradiotherapy.

## Introduction

Cervical cancer is the fourth most prevalent malignancy in women, with an estimated 569,800 new cases and 311,300 deaths worldwide in 2018 1, 2. Approximately 20-22% of cervical cancer cases relapse within 5 years of treatment, and its prognosis depends on treatment effectiveness 3. For instance, cases that have a high risk of relapse because of adverse pathologic factors are effectively treated by undergoing a radical hysterectomy plus adjuvant chemoradiotherapy 4, 5. Early identification of those that do or do not respond well to treatment could be used to optimize therapeutic strategies that eliminate the tumor while minimizing treatment and decreasing toxicity, which is a step toward individualized therapy.

The adaptive and innate immune responses participate in tumor immunosurveillance and cancer development 6, 7. Immune cells that migrate from the blood into the tumor, e.g., T-cells, B-cells, natural killer (NK) cells, or macrophages, are defined as tumor-infiltrating immune cells. Interestingly, tumor-infiltrating immune cells are commonly found in the tumor stroma or intraepithelium. As such, tumor-infiltrating immune cells are the main players in facilitating the immune response against cancer, particularly as their levels can predict therapeutic effectiveness and survival. CD3, CD4, and CD8 are common T cell markers, but each serves their own function under normal conditions. For instance, CD3+ T cells are markers for all T cells. CD4+ T cells are also called T helper (Th) cells, whereas mature CD8+ T cells are termed cytotoxic T lymphocytes (CTLs). Of note, CD3+ T cells represent all total T lymphocytes, whereas CD4+ T and CD8+ T cells represent the relative composition of their subgroups (T helper (Th) and cytotoxic T lymphocytes (CTL), respectively. Tumor infiltration by CD4+ T cells has been associated with poor relapse-free survival in translocation renal cell carcinoma (RCC) 8. High densities of CD8+ T cells are associated with poor prognosis in prostate cancer 9, clear cell RCC 10, 11, Hodgkin lymphoma 12, and follicular lymphoma 13. In a previous breast cancer study, patients with high numbers of CD8+ T cells within the invasive margins (IMs) of tumors had a poor prognosis 14. However, CD8+ T cell densities in the tumor center (TC) were not associated with breast cancer prognosis 14. In short, CD8+ T cells can provide contradictory prognostic predictions in different regions of tumors 14. In addition, there has been no consensus on the roles of B cells (CD20+) and NK cells (CD57+) in cancer 15. Tumor-associated macrophages (TAMs) are important immune cells in the tumor microenvironment. TAMs can be divided into two main groups: M1 and M2 16-18. CD68 is a pan-macrophage marker expressed in both MI and M2 macrophages, whereas CD163 is specifically expressed in M2 macrophages. Increased density of CD163+ macrophages corresponds with a worse prognosis in patients with cervical 19, ovarian 20, 21, breast, and bladder cancers 22, 23. Taken together, these results suggest that density patterns of different immune cell markers (namely CD3, CD4, CD8, CD20, CD57, CD68, and CD163) could be used to predict the prognosis of patients with cervical cancer and aid in evaluating the effectiveness of adjuvant chemoradiotherapy.

In this study, we evaluated whether TC-infiltrating immune cells in tissue specimens, taken before treatment regimens, act as predictive or prognostic biomarkers for patients with cervical cancer. We performed immunohistochemistry (IHC) on tissue microarrays containing patient specimens to visualize and quantify immune cells in the TC and generated an immune cell model to predict the prognosis of cervical cancer and the effectiveness of adjuvant chemoradiotherapy.

## Materials and Methods

### Patient selection

**Figure 1:**
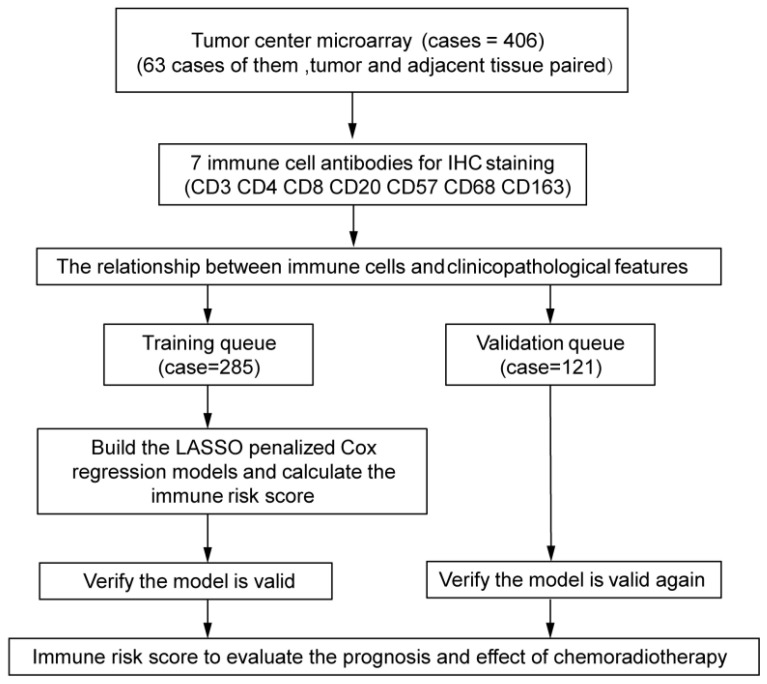
**Study design** Assessment of immune cell densities in the adjacent tissue (n=63), tumor center (TC; n=406). TC microarray data were used to visualise immune cell distributions and analyse correlations between immune cell levels, clinicopathological characteristics, and prognosis. Based on this analysis, an immune cell model was generated to predict the effects of adjuvant chemoradiotherapy.

The experimental design is shown in Figure 1. The independent dataset included 406 patients with cervical cancer who underwent surgery between 2015 and 2018 at the Second Affiliated Hospital and the First Affiliated Hospital of Wenzhou Medical University. Tumor center tissues were collected from 406 patients. Inaddition, 63 paired adjacent tissues were collected from all the patients. The patients were randomly divided into training (*n* = 285) and validation (*n* = 121) queues. The clinicopathological characteristics of the patients are shown in Tables 1 and 2. Patients were followed-up every 3 months for the first year and every 6 months thereafter.

All patients provided written informed consent before participation. Inclusion criteria included the following: (1) informed consent; (2) The surgery or biopsy was performed before chemotherapy (CT) or radiotherapy (RT); (3) complete follow-up data; (4) stage ≥ cervical carcinoma *in situ* (CIS); and (5) diagnosis confirmed by histopathology according to the 2009 International Federation of Gynecology and Obstetrics staging system.

Overall survival (OS) was calculated as the time from the diagnosis to death by any cause. The median follow-up time in the training and validation queues was 43.47 and 43.48 months, respectively, and 21 and 8 patients died in the two queues, respectively. All 381 patients in stages CIS-IIA2 elected to undergo surgery first. There were 18 patients in stage IIB, 10 of whom were directly treated with concurrent chemotherapy and radiotherapy (CRT), whereas 8 patients had the surgery first. Of the seven patients in stage III-IV, three were treated with CRT, three with CT or RT, and one underwent surgery at another hospital before receiving CRT. In the statistical description, stages were divided into CIS+I, IIA1, IIA2, IIB, and III-IV groups. The stages were divided into three groups (IB1+IIA1, IB2+IIA2, and IIB-IV) based on tumor size and similarity of treatment; CIS-IA2 patients who have not received adjuvant CRT were excluded. The study was performed in accordance with the International Ethical Guidelines for Biomedical Research Involving Human Subjects and was approved by the Institutional Review Board of the Second Affiliated Hospital of Wenzhou Medical University.

### IHC and evaluation of immunostaining

Different tissue areas were located using hematoxylin and eosin staining (Figure S1B). To collect the TC, 406 patients tissue cores of 1.5 mm that were then used to generate the tumor core tissue chip. Tissue cores of 1.5 mm that from the 63 paired paracancerous tissues were used to make adjacent tissue chips (negative control; NC). The tissue microarrays were incubated at 60 °C for 2 h, dewaxed with xylene, and hydrated using an alcohol gradient. Antigen retrieval was performed using the high-pressure repair method. Endogenous peroxidase activity was blocked with 0.3% H_2_O_2_. After blocking in goat serum for 10 min, the microarrays were incubated overnight at 4°C with primary antibodies against CD3 (Clone F7.2.38, cat. no. A0452; dilution 1:100; Dako, Glostrup, Denmark), CD4 (clone 4B12, cat. no. IR649; dilution 1:100; Dako), CD8 (clone C8/144B, cat. no. IR623; dilution 1:150; Dako), CD20 (clone L26, cat. no. IR604; dilution 1:150; Dako), CD57 (clone TB01, cat. no. ab87274; dilution 1:100; Abcam, Cambridge, UK), CD68 (clone OT14G1, cat. no. Kit-0026; MXB Biotechnologies, Inc., Fujian, China), and CD163 (clone 10D6, cat. no. ZM-0428; ZSGB-BIO, Beijing, China). After washing with phosphate-buffered saline, the microarrays were incubated with horseradish peroxidase-conjugated anti-rat/rabbit IgG (cat. no. K5007; Dako) for 10 min at 22 °C. Finally, the microarrays were stained with a 3,3-diaminobenzidine solution (dilution 1:50) for 5 s while under observation through a microscope, and hematoxylin was used to counterstain the nuclei.

Slides were analyzed on a Pannoramic MIDI automatic digital slide scanner (3DHISTECH, Budapest, Hungary) with QuantCenter analysis software (3D HISTECH). The QuantCenter feature NuclearQuant was used to automatically identify nuclei and score dark brown, brown-yellow, light yellow, and blue nuclei as strongly positive, moderately positive, weakly positive, and negative, respectively. The number and percentage of cells in each category in each tissue point were used to calculate H-scores, using the formula as follows: H-score=∑(*PI* × *I*) = (percentage of cells of weak intensity × 1) + (percentage of cells of moderate intensity × 2) + (percentage of cells of strong intensity × 3) 24, 25. Two pathologists who were blinded to the clinicopathological data independently evaluated the results.

### Statistical analysis

Statistical analysis was performed in Stata (version 15; StataCorp, College Station, TX, USA), and R, version 3.6.1 (The R Foundation for Statistical Computing, http://www.r-project.org/). The Cox regression model with the least absolute shrinkage and selection operator (lasso) penalty was used on the training queue to determine the optimal β coefficient of the prognostic feature and the likelihood deviance, using the “glmnet” package in R. The formula was as follows: Risk score = Σβi × expGenei. Plotted statistical values (paired-boxplot, correlation matrix diagram, Kaplan-Meier survival analysis, receiver operating characteristics, Calibration, Heatmap, violin plot, boxplot and scatter plot) were applied using R with packages including the following: ggplot2, cowplot, GGally, survival, dplyr, tidyr, survminer, dplyr, pheatmap, cowplot, timeROC, tibble, survivalROC, tidyverse, rms, ggpubr, ggstatsplot. The median (range) was used for continuous variables with an abnormal distribution, and comparisons among the different groups were made using the Kruskal-Wallis test (Table 1 and 2). Univariate and multivariate Cox regression analyses indicated that p < 0.05 was statistically significant (Table 3). Categorical variables were described by counts and percentages, and comparisons among the different groups were made using Fisher's exact test or Pearson's chi-squared test (Table 4). *P* < 0·05 was considered statistically significant.

## Results

### Characteristics of infiltrating immune cells in cervical cancer center

**Figure 2:**
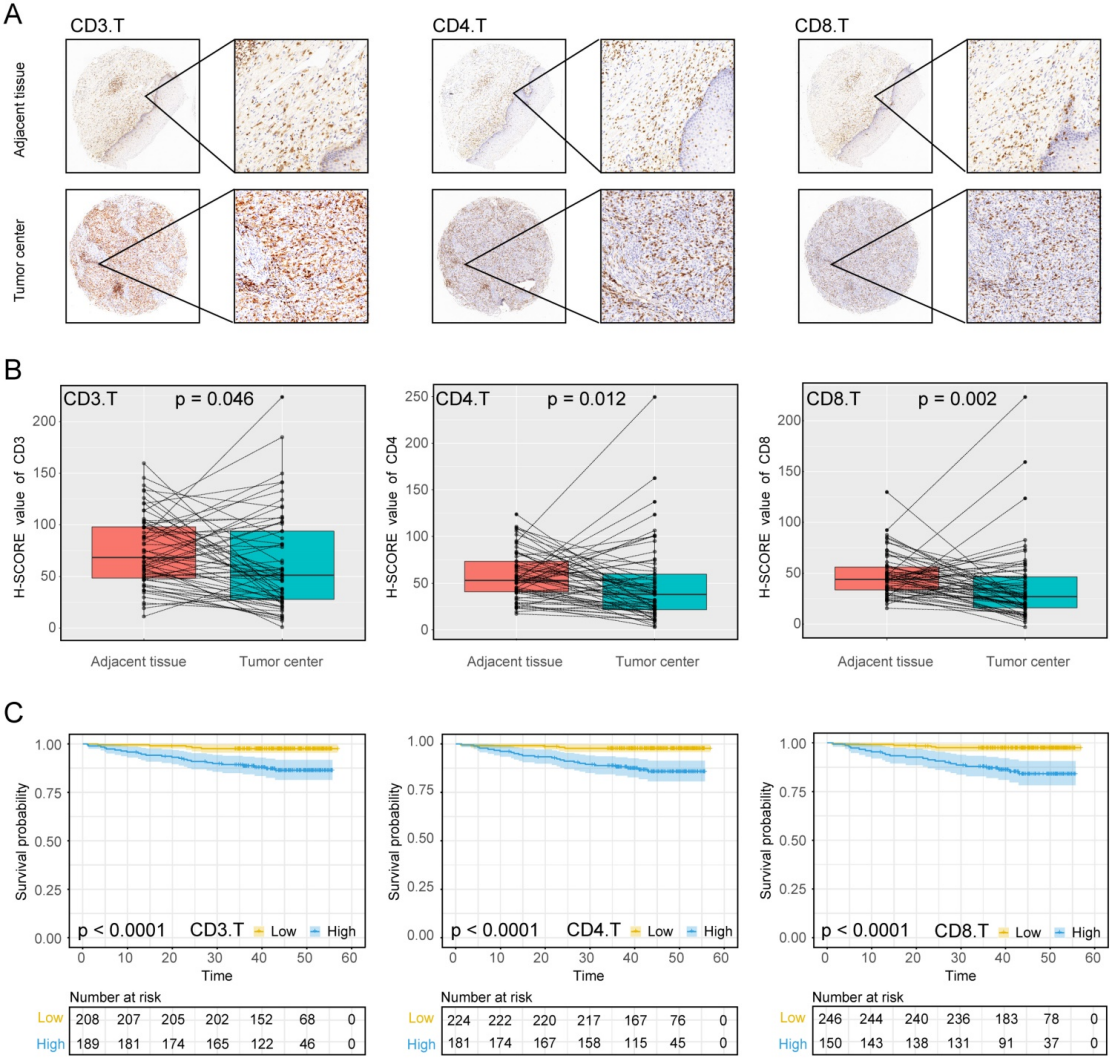
**Expression of T cell markers in cervical cancer** A, Tumour centre and adjacent tissue microarrays were stained for CD3, CD4 and CD8. B, Paired box plot of H-SCORE value. C, KM survival analysis of tumor centers.

**Figure 3:**
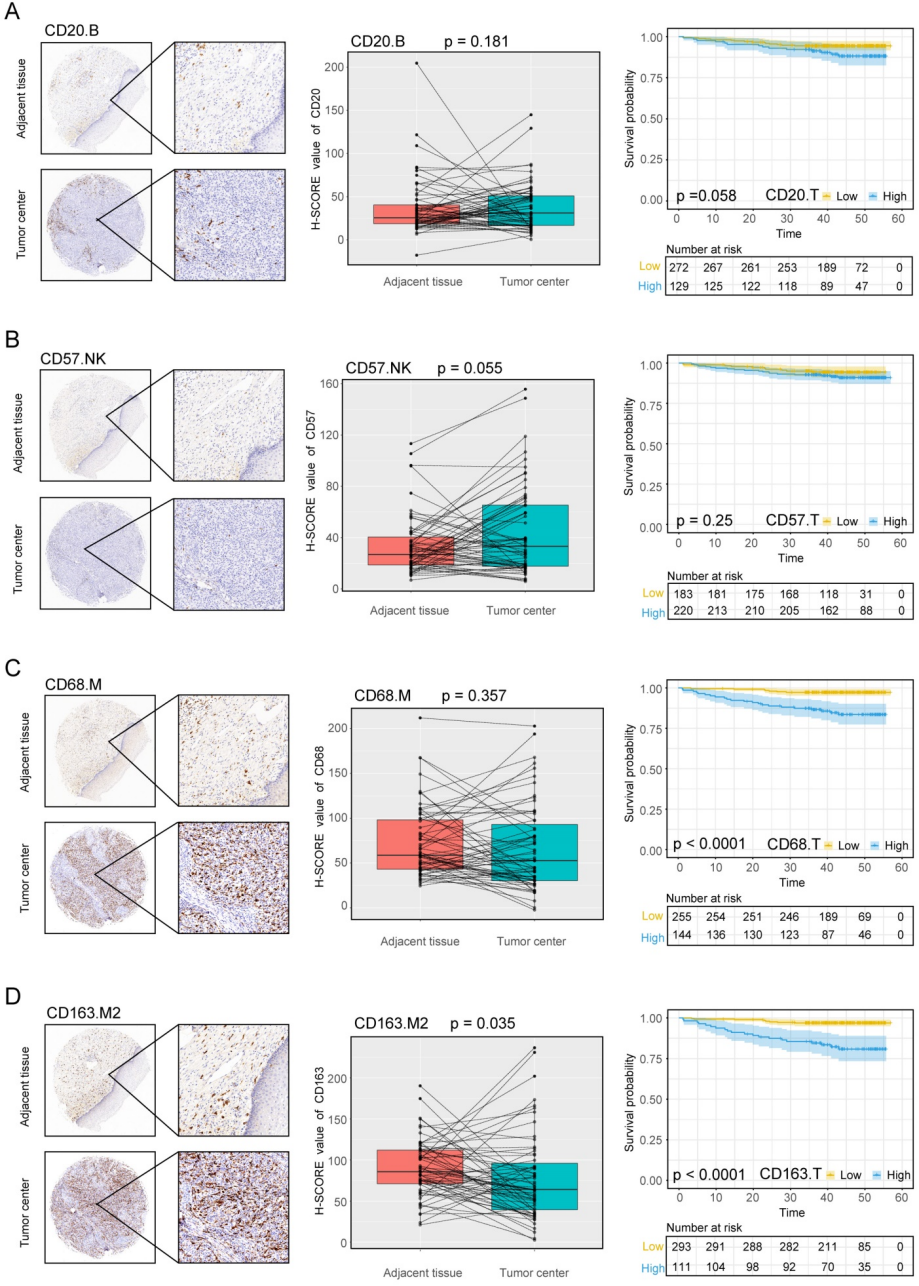
**Expression of the B cell marker CD20, the natural killer cell marker CD57, and macrophage markers in cervical cancer** A, B, C, D, CD20, CD57, CD68, CD163 expression levels in the tumor center and adjacent tissue microarrays; box plot of paired samples; KM survival analysis.

**Figure 4:**
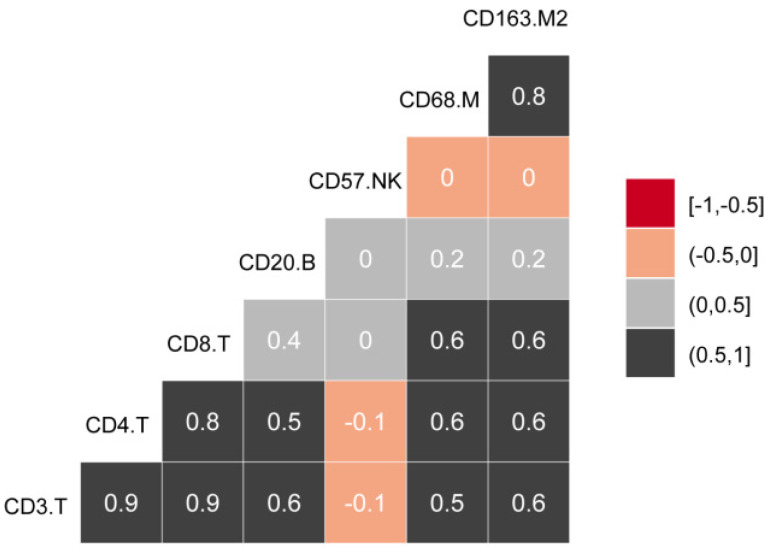
Correlations between infiltrating immune cell types in cervical cancer.

The experimental process is shown in Figure 1. Tumor microarrays, which contained 406 TC samples and 63 paired adjacent tissues samples, were stained for T cells, NK cells, and macrophages (Figure 2 and 3). The mean H-scores of CD3+ T cells, CD4+ T cells, CD8+ T cells, CD20+ B cells, CD57+ NK cells, CD68+ M cells, and CD163+ M2 cells in the TC were 75.323, 57.461, 38.680, 29.709, 26.859, 51.193, and 69.097, respectively (Figure S1A). The H-scores of CD3+ T cells, CD4+ T cells, CD8+ T cells, and CD163+ M2 cells were considerably higher in the adjacent tissue than in the TC, whereas the levels of CD57+ NK cells were elevated in the TC. The higher the densities of CD3+ T cells, CD4+ T cells, CD8+ T cells, CD68+ M cells, and CD163+ M2 cells, the prognosis significantly worsened (Figure 2 and 3). Therefore, we used the TC microarray data in the prediction model. Among the markers, CD3+ T cells had the highest mean expression in the TC, followed by CD163+ M2 cells; CD57+ NK cells had the lowest mean expression (Figure S1A). Importantly, our data showed that CD3+CD4+ (double positive) T cells, that is, containing both total T and Th cells, were predominant. We performed correlation analysis between the seven immune cell markers in the TC. The correlation coefficients between CD3+ T cells, CD4+ T cells, and CD8+ T cells ranged from 0.8 to 0.9 (Figure 4), indicating significant positive correlations among them. The correlation coefficient between CD68+ M cells and CD163+ M2 cells was 0.8, also an evident positive correlation (Figure 4); moreover, CD68+CD163+ double positive M2 macrophages were also numerous in the TC from cervical cancer tissues. These results mean that the function and role of T cells can be altered following tumor infiltration. Infiltrating T cells and macrophages in TC of cervical cancer were associated with poor prognosis.

### Associations between H-scores, clinicopathological characteristics, and prognosis

Immune cell densities in the TC microarray samples with different clinicopathological characteristics are shown in Tables 1 and 2. The densities of CD3+ T cells, CD8+ T cells, and CD163+ M2 cells were higher in squamous cell carcinoma (SCC) than in non-SCC types (*P* < 0.005, *P* < 0.037, and *P* < 0.025, respectively), whereas CD57+ cells were increased in non-SCC cervical cancer (*P* < 0.011) compared with SCC. CD3+ T cells, CD4+ T cells, CD8+ T cells, CD68+ M cells, and CD163+ M2 cells were highly prevalent in stages IIA1, IIA2, and IIB than in stages CIS+I and III-IV (*P* < 0.001, *P* < 0.001, *P* < 0.002, *P* < 0.001, and *P* < 0.001, respectively). As the stage increased, CD20+ B cells and CD57+ NK cells decreased (*P* < 0.001 for both cell types). In samples with poor differentiation and lymph node involvement, CD3+ T cells, CD4+ T cells, CD8+ T cells, CD68+ M cells, and CD163+ M2 cells significantly increased, whereas CD57+ NK cells significantly decreased in the same environment (*P* < 0.003, *P* < 0.001, *P* < 0.038, *P* < 0.001, *P* < 0.001, and *P* = 0.014, respectively). CD57+ NK cells displayed significantly higher infiltration in younger patients (≤57.5 years; *P* < 0.010); however, none of the other cell subsets correlated with age.

To evaluate whether the levels of different immune cell subsets influenced the success of treatment regimens and patient prognosis, we divided the patients into three groups based on their treatment plans: follow-up without RT or CT (non-CRT), RT or CT, or concurrent RT and CT (CRT). The infiltration of CD3+ T cells, CD68+ M cells, and CD163+ M2 cells increased significantly in CRT patients compared with non-CRT patients (all *P* < 0.010). CD20+ B cells and CD57+ NK cells displayed the opposite trend, with significantly higher expression in non-CRT patients than in those treated with CRT (both *P* < 0.010). Next, we analyzed the correlations among immune cell types, adjuvant therapies, and patient prognosis. CD3+ T cells, CD4+ T cells, CD8+ T cells, CD20+ B cells, CD68+ M cells and CD163+ M2 cells showed significant differences in the CRT group, with higher densities in deceased patients (Figure S2). In the RT or CT group, CD163+ cells were significantly higher in the deceased patients than in the living patients (Figure S2). Alternatively, in the non-CRT group, the densities of all infiltrating immune cells did not differ between deceased and living patients (Figure S2).

Using the data provided from the patients' characteristics (Tables 1 and 2), we generated a scatter plot to visualize the differences among patients, particularly those living or deceased with the densities of immune cells. As shown in Figure S3, deceased patients had more infiltration of CD3+ T cells, CD4+ T cells, and CD8+ T cells, CD68+ M cells, and CD163+ M2 cells. We also analyzed correlations between immune cell markers and survival prognosis (Figures 2 and 3). CD3+ T cells, CD4+ T cells, CD8+ T cells, CD68+ M cells, and CD163+ M2 cells infiltration of the TC were significantly correlated with poor prognosis regardless of the stage and other clinical characteristics (*P* < 0.001). These data indicate that the numbers of CD3+CD4+CD8+ (triple positive) T cells and CD68+CD163+ (double positive) M2 cells corresponded to a poor prognosis.

### Construction of the tumor immune model

**Figure 5:**
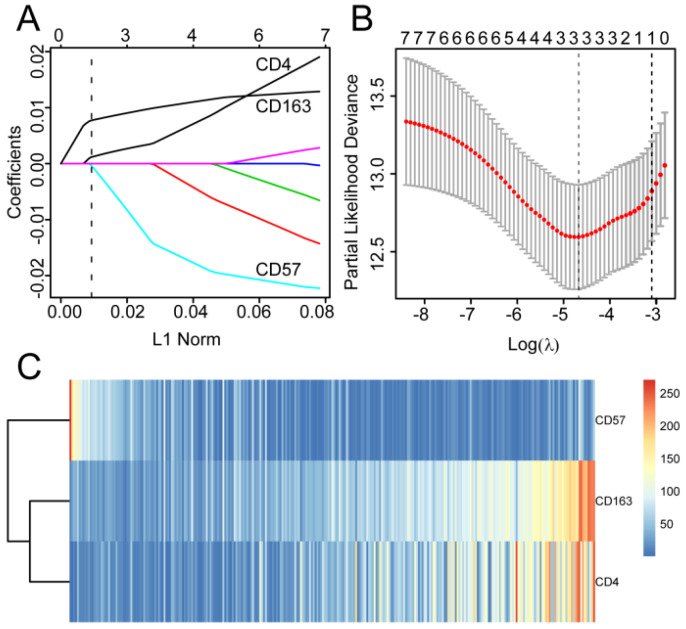
**Modelling in the training queue** A, Partial likelihood deviance values for lasso-penalised Cox regression coefficient profiles. The right dotted vertical line represents the lambda.lse partial likelihood deviance. The left light dotted vertical line at the value log lambda = -4.7 was chosen by ten-fold cross-validation to represent the partial likelihood deviance. B, Lasso-penalised Cox regression coefficient profiles of the selected tumour immune features. The dotted vertical line represents the value lambda.min = 0.009312408. C, Heatmap of the expression of the three selected immune cell types. Red and blue represent high and low expression, respectively.

The 406 patients were randomly divided into training and validation queues in silico at a 7:3 ratio. Using the training set, the seven immune cell types were used to construct a TC tumor immune risk score via lasso-penalized Cox regression analysis. Using this analysis, the three immune cell subsets with the most significant prediction features included CD4+ T cell, CD57+ NK cell, and CD163+ M2 cells, and their coefficients were calculated using log lambda = -4.7 (Figure 5A, 5B). The partial likelihood deviance was 12·59382 (Figure 5B). A regression formula was generated based on the three TC immune cell infiltration features: immune risk score = 0.002839642 × CD4+TTC + 0.009183710 × CD163+M2TC - 0.009250865 × CD57+NKTC. Risk scores ranged from -2.4 to 2.3. To assess the prognostic value of the tumor immune risk score as a linear variable, we plotted smooth immune risk score curves of OS in the training and validation queues (Figure S4 and S5). Patients were considered low- or high-risk when their tumor immune risk scores were either <0.87 or ≥0.87, respectively. The low- and high-risk patient numbers in the training queue were 205 and 80, respectively. In the validation queue, the low- and high-risk patient numbers were 89 and 32, respectively.

### Verifying model validity

**Figure 6:**
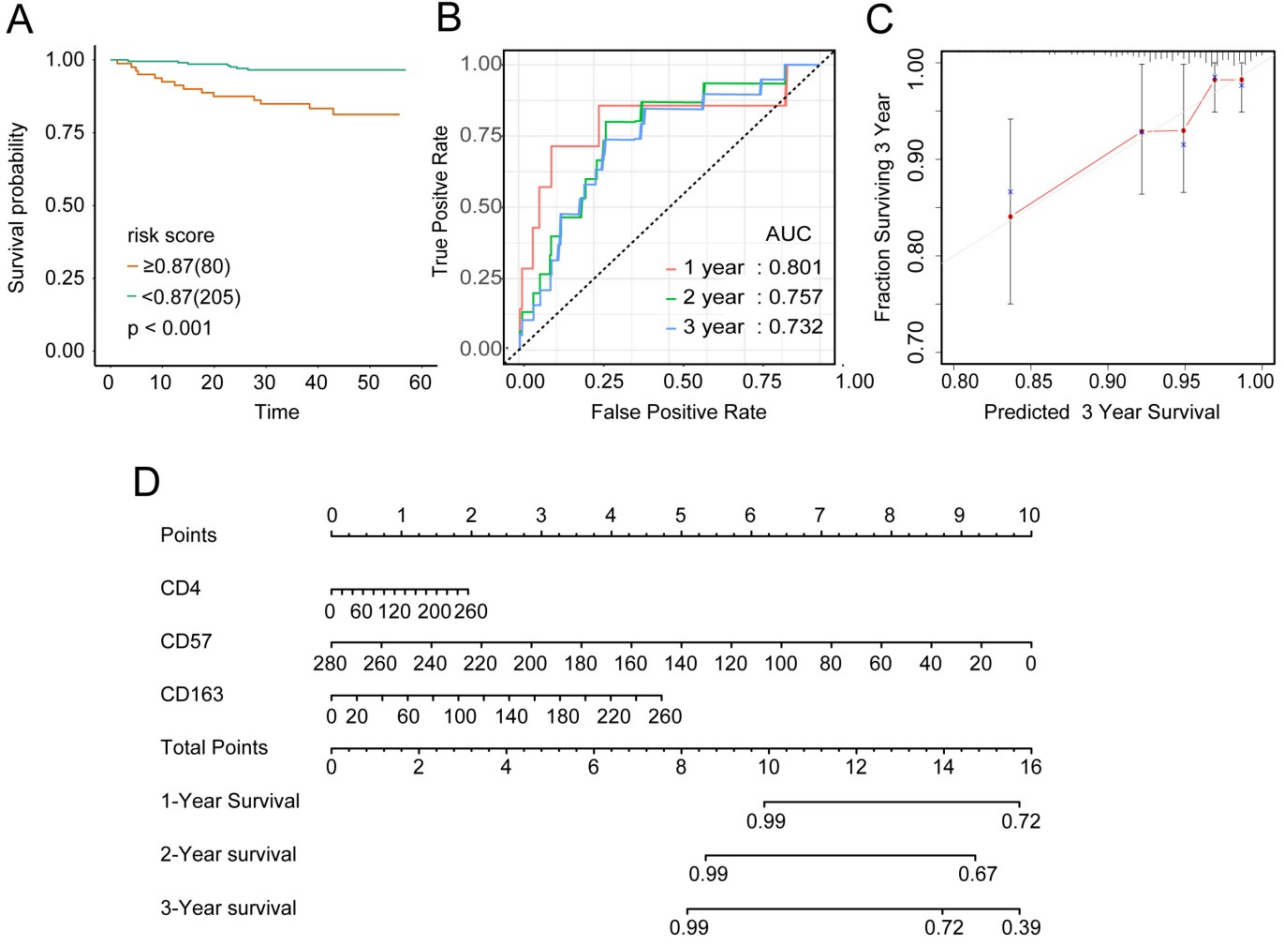
**Verification of model validity using the training queue** A, Partial likelihood deviance values for lasso-penalised Cox regression coefficient profiles. The right dotted vertical line represents the lambda.lse partial likelihood deviance. The left light dotted vertical line at the value log lambda = -4.7 was chosen by ten-fold cross-validation to represent the partial likelihood deviance. B, Lasso-penalised Cox regression coefficient profiles of the selected tumour immune features. The dotted vertical line represents the value lambda.min = 0.009312408. C, Heatmap of the expression of the three selected immune cell types. Red and blue represent high and low expression, respectively.

**Figure 7:**
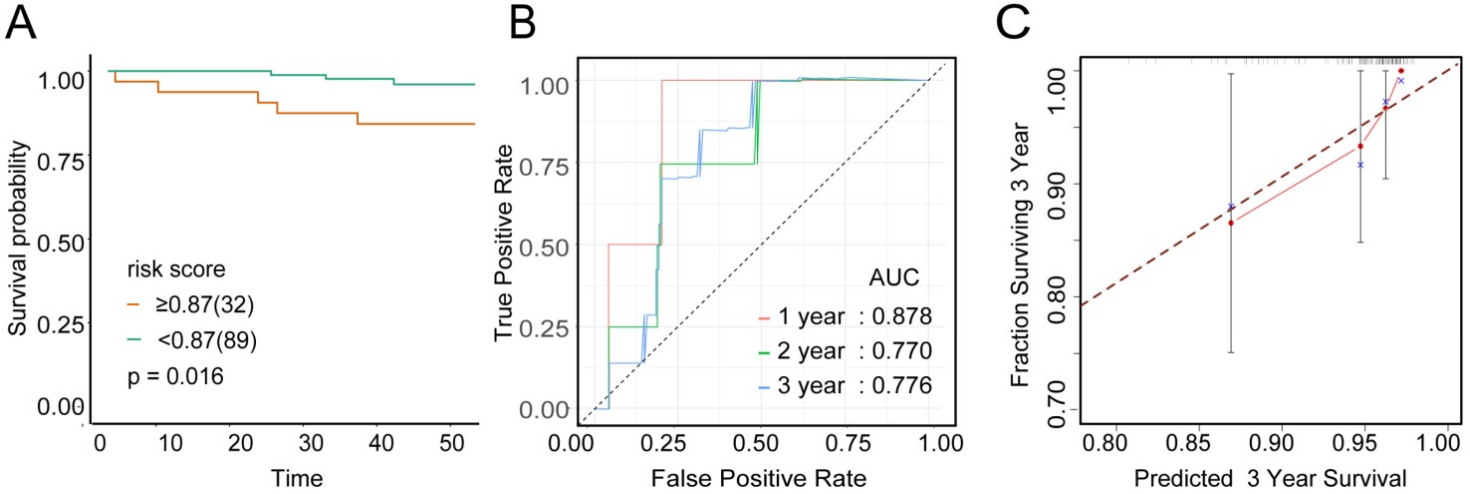
**Verification of model validity using the validation queue** A, Kaplan-Meier survival analysis of patients with cervical cancer in the validation queue, based on their tumour immune risk scores. B, ROC analysis of the tumour immune model in predicting the 1-, 2-, and 3-year OS of patients in the validation queue. C, Calibration analysis of the tumour immune model in the validation queue.

To examine the rationality of our tumor immune model, we performed hierarchical clustering using immune cell clusters taken from the training and validation queues (Figure 5C and S5B). To examine the prognostic value of the tumor immune model, we compared the OS among various patient groups using Kaplan-Meier survival analysis. In both queues, CD4+^high^CD163+^high^CD57+^low^ patients had significantly worse OS than CD4+^low^CD163+^low^CD57+^high^ patients (*P* < 0.001 and *P* < 0.016, respectively; Figures 6A and 7A). The receiver operating characteristic (ROC) values over time were 0.801 (1 year), 0.757 (2 years), and 0.732 (3 years) in the training queue versus 0.878 (1 year), 0.770 (2 years), and 0.776 (3 years) in the validation queue (Figure 6B and 7B). Calibration analysis of the model demonstrated good agreement between prediction and observation in the training and validation queues (Figure 6C and 7C). A nomogram was developed to predict the 12-, 24-, and 36-month probability of OS in patients in the training queue. The nomogram was generated based on CD4+ T cell, CD57+ NK cell, and CD163+ M2 cell densities, which was also used to further verify the feasibility of the model (Figure 6D). Concordance indices for the prediction model were 0.74 (standard error: 0.052) and 0·764 (standard error: 0.072) for the training and validation queues, respectively.

### Immune risk score independently predicts survival

The distributions of clinical characteristics in patients with high- and low-tumor immune risk scores were significantly associated with status, stages, differentiation, lymph gland, chemoradiotherapy and age (*P* < 0.05, Table 4). The Cox proportional hazard model was used to examine hazard ratios (HR) and 95% confidence intervals (CIs). Through univariate Cox regression analysis (HR: 5.471, 95% CI: 2.543-11.768; *P* = 0.001; Table 3), the immune risk score was determined to be significant. Univariate analysis also showed that the variables associated with OS included stages (HR: 4.444, 6.049, 11.368, 22.014; *P* = 0.002, 0.004, 0.001, 0.001), differentiation (HR: 3.458; *P*= 0.008), lymph gland (HR: 7.177; *P* = 0.001), chemoradiotherapy (HR: 4.510; *P* = 0.001), and age (HR: 2.528; *P* = 0.013). After adjusting for the clinical variables, multivariate Cox regression analysis was performed. These results showed that our tumor immune risk score remained an independent and powerful prognostic factor for OS for overall queue (HR: 4.340, 95% CI 1.894-9.943; *P* = 0.001; Table 3). Moreover, Cox regression analysis also showed that stages (HR: 8.857, 25.237; *P* = 0.002, 0.001) and lymph gland (HR: 2.765; *P* = 0.027) were independent factors in determining prognosis.

### The TC immune model can predict the effects of adjuvant chemoradiotherapy on cervical cancer

**Figure 8:**
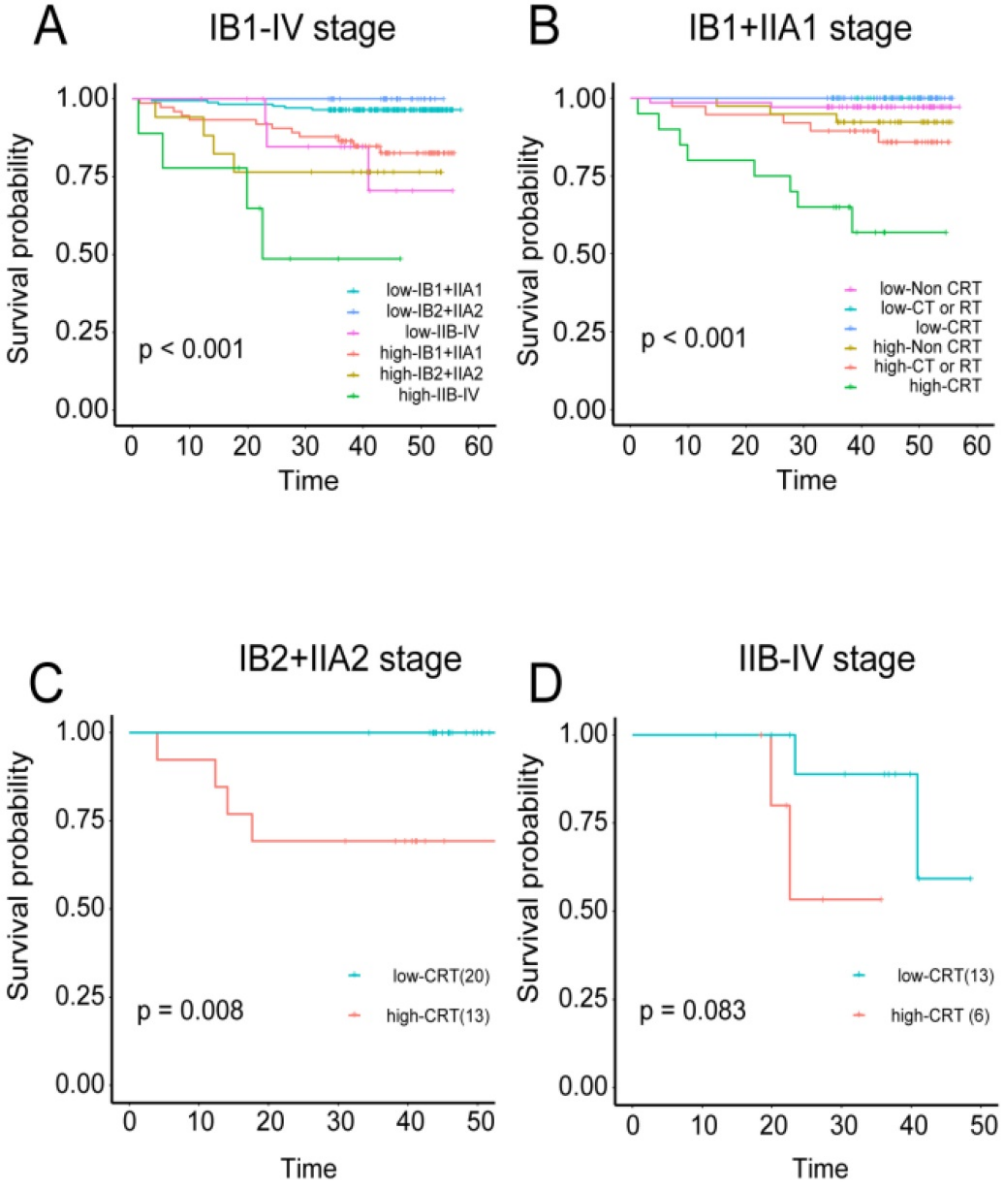
** Predictive value of the immune risk score in different stages and with different treatment regimens** A, Patients in three different stage groups were divided into high- and low-immune risk score groups. B, Patients in stage IB1+IIA1 were divided into three groups according to their treatment regimens (non-CRT, CT or RT, and CRT), and each group was stratified by immune cell risk score. C, Stage IB2+IIA2 patients treated with CRT were stratified by immune cell risk score. D, Stage IIB-IV patients treated with CRT were stratified by immune cell risk score.

The correlations were examined among densities of immune cell subset, adjuvant therapy, and cervical cancer prognosis. Regardless of the training or validation queue, patients with high tumor immune model scores had significantly worse OS in stages IB1+IIA1, IB2+IIA2, and IIB-IV (independently: *P* < 0.001, *P* < 0.0008, and *P* < 0.044 respectively; combined: *P* < 0.001; Figure S6A-C and 8A). Patients in stage IB1+IIA1 were also divided into three categories based on their treatment regimens (i.e., non-CRT, CT or RT, and CRT). High-scoring patients in the CT or RT and CRT groups had worse OS than those with low scores (*P* = 0.03 and *P* < 0.001, respectively; combined *P* < 0.001; Figure S6E, S6F, and 8B). For patients in stage IIB2+IIA2, those in the CRT group also displayed this trend, with more favorable prognosis among patients with low-risk scores (*P* = 0.008; Figure 8C).

## Discussion

We explored the characteristics of the immune cells infiltrating cervical cancer by analyzing immune cell area distributions and correlation with clinical features. CD3+ T cells, CD4+ T cells, and CD8+ T cells were strongly correlated, and CD3+CD4+ (double positive) T cells were predominant in the TC. Increased CD3+ T cells, CD4+ T cells, and CD8+ T cells in the TC were unexpectedly correlated with poor prognosis. With progression in differentiation type and lymph node metastasis, the amount of CD3+ T cells, CD4+ T cells, and CD8+ T cells gradually increased. These immune cells are also associated with poor prognosis in head and neck cancer 26, breast cancer 27-29, prostate cancer 9, 30, melanoma 31, colorectal cancer 11, 32. The mesenchymal subtype of colorectal cancer is densely infiltrated by CD8+ T cells, which is an indicator of a poor prognosis. Also in this subtype, cancer-associated fibroblasts may promote inflammation, angiogenesis, and metastasis, thereby repressing the antitumor activity of CD8+ T cells while fueling vascular regeneration, tumor growth, and stromal rebuilding 32. Notably, CD8+ T cells function in cervical cancer may also be indirectly inhibited by fibroblasts, resulting in poor prognosis.

There are two possible outcomes of T cells infiltrating the TC. One possibility could be that the immune cells attempt to clear the tumor cells from the region. Alternatively, immune escape occurs, and although the number of immune cells infiltrating the TC increases, their phagocytic capabilities are diminished. Immune cells that infiltrate the TC directly interact with tumor cells, thereby reflecting the function of infiltrating immune cells within the tumor microenvironment. Therefore, we focused on infiltrating immune cells in the TC in this study.

There have been similar reports on the accumulation of CD8+ T cells in cervical cancer; however, their function was inhibited in various ways 33. One study suggested that exposure of CD8+ T cells to human papillomavirus (HPV)-infected epithelial cells may leave them unable to clear antigen-consistent, HPV-infected tumor cells 34. Immune escape mechanisms can also invalidate memory CD8+ T cells 35. Lastly, downregulation of major histocompatibility complex I antigen presentation mechanisms in HPV-infected epithelial cells can affect CD8+ T cell recognition and clearance 34, 36, 37. Based on our results, we found that the T cells in the IM exerted antitumor effects. Upon initial infiltration of the TC, they have powerful antitumor functions and effectively control the tumor. However, over time, the tumor cells evade CD4+ T cell immune surveillance by modifying their own surface antigens. This change in the microenvironment decreases or neutralizes the immune response exerted by the T cells, thereby reducing the body's antitumor defenses 38, 39.

The density of CD20+ B cells did not differ between the TC and adjacent tissue; moreover, these cells did not correlate with survival prognosis. A previous study reported 5-year OS rates of 42% and 36% in patients with cervical cancer with and without CD20+ B cell infiltration, respectively 40. These rates were not significantly different, a finding that is consistent with our results. However, for other immune cell types, such as NK cells, their role in cervical cancer remains unclear. Infection by high-risk HPV causes cervical cancer via its oncoprotein, HPV16 E6/E7, inhibits interleukin 18-induced interferon-γ production in NK cells, which may promote viral pathogenesis 41. However, in this study, high levels of CD57+ cell infiltration was observed in patients with non-SCC malignancies, early stage malignancies, high differentiation, no lymph nodemetastasis, no CRT, and those under 57.5 years of age. These data suggest that CD57+ NK cells positively correlate with clinical indicators of good prognosis, suggesting that these cells serve a protective role.

Macrophages are an important component of the innate immune system, and they function by phagocytosing pathogens, presenting antigens, and killing tumor cells. However, macrophages that infiltrate the tumor microenvironment promote the occurrence and development of tumors. The density of CD163+ M2 cells was higher in the adjacent tissue than in the TC, whereas CD68+ M cells did not differ between the regions in the current study. However, CD68+CD163+ (double positive) macrophages were numerous in the TC from cervical cancer tissues and the increased densities of both markers were associated with poor prognosis. CD163+ M2 macrophages fuel the growth and development of most cancers; moreover, increased numbers are associated with negative prognosis in breast, bladder, ovarian, gastric, and prostate cancers as well as RCC and melanoma 42. M2 macrophages, which are alternatively activated, sabotage immunity by producing transforming growth factor-β, interleukin-10, prostaglandin E2, and C-C motif chemokine ligand 22 43-45. Numerous studies have demonstrated that TAMs stimulate tumor growth and progression and are significantly associated with unfavorable prognosis in various malignancies 46. Consistent with our results, expression of CD163+ M2 cells in tumors expression was predictive of negative clinical outcomes in 106 patients with head and neck SCC after definitive CRT 47. Stromal cells, including immune cells, are a major component of the tumor microenvironment, and TAMs play important roles in this environment. Interactions between TAMs and tumor cells promote the inflammatory response, leading to tumor progression 48. Notably, M2 cells induce immune tolerance and promote tumor progression. M2 cells were highly expressed in the TC, which suggests that they are factors that promote tumor progression in cervical cancer.

Our patients displayed tremendous heterogeneity in clinical prognosis, even with similar pathological types, stages, levels of differentiation and lymph node involvement, chemoradiotherapy administration, and ages. However, we found that the levels of CD3+ T cells, CD4+ T cells, CD8+ T cells, CD20+ B cells, CD57+ NK cells, CD68+ M cells, and CD163+ M2 cells could significantly improve prognosis prediction based on clinical characteristics. A recent study proposed that immune cell analysis could result in prognostic tools that are independent of clinical characteristics 49.

In 1997, Robert Tibshirani first proposed the lasso method for variable selection and shrinkage in Cox's proportional hazards model, which is more accurate than stepwise selection and is widely used in clinical prediction models 50. Through a similar approach, we built an immune prediction model and to estimate the tumor immune risk score. In the optimal model, increased CD4+ T cells, CD57+ NK cells, and CD163+ M2 cells in the TC indicated poor prognosis. Kaplan-Meier, ROC, and calibration analyses were performed on the training and validation queues, and a nomogram was generated using the training queue. The tumor immune risk score was also analyzed for correlations with clinical characteristics, which in turn verified the predictive ability of the lasso-penalized Cox method.

Additionally, by dividing the patients into high- and low-immune risk score groups, we observed that the prognostic value of the model as low-scoring patients (CD4+^low^CD163+^low^CD57+^high^) had better 3-year OS than high-scoring patients (CD4+^high^CD163+^high^CD57+^low^). Based on our univariate and multivariate Cox regression analyses, the tumor immune risk score was an independent and powerful outcome indicator. Therefore, the tumor immune risk score generated using this model is a meaningful predictor of cervical cancer prognosis.

Pathological staging is the most commonly used clinical prognostic indicator; however, patients in the same stage can have different prognoses. We supplemented the prognostic evaluation system with the immune risk score. This approach can not only predict prognosis independently, but also classify it according to the infiltration levels of immune cells based on the pathological stage and significantly improve the accuracy of prognosis prediction at each stage. When patients in stages IB1+IIA1, IB2+IIA2, and IIB-IV in the overall dataset were stratified according to their immune risk score, those with the higher scores had worse prognosis; conversely, those with lower scores had better prognosis. The differences among the stages were also significant.

Interestingly, the immune risk score was also able to predict the effects of CT or RT. When patients in stages IB1+IIA1, IB2+IIA2, and IIB-IV in the overall dataset were classified into non-CRT, CT or RT, and CRT groups and stratified via their immune risk scores, patients in the CRT group with high immune risk scores indicated a poor prognosis, whereas those with low immune risk scores were favorable. The differences were statistically significant for patients in stages IB1+IIA1 and IB2+IIA2, and the same trend was observed for those in stage IIB-IV, although the difference was not significant.

In the CRT treatment group, synchronous CRT was very effective in patients with low immune risk scores but performed poorly in patients with high immune risk scores. These data suggest that for patients with high immune risk scores, the disadvantages of CRT therapy may outweigh the benefits. For these types of patients, more effective or alternative treatments should be sought, such as immunotherapy options.

In conclusion, we have systematically performed IHC-based analysis of immune cells that infiltrated the TC in cervical cancer samples, analyzed correlations among T cells, NK cells, B cells, macrophages, and clinical features. Additionally, we described markedly different functions of T cells and macrophages following their infiltration to different regions in the tumor. Through this approach, we constructed an immune cell-based predictive model that can effectively predict survival and the effects of CT and RT. Clinical application of this model could help identify patients who are not suitable for CT and RT regimens and aid in screening out patients suitable for immunotherapy.

## Supplementary Material

Supplementary figures.

## Figures and Tables

**Table 1:** Description of univariate in clinicopathological features

Factor	N	CD3 median (IQR)	*P* value	CD4 median (IQR)	*P* value	CD8 median (IQR)	*P* value
**Pathological type**							
SCC	355	41.6 (17.0, 82.9)	0.11	69.5 (28.3, 113.4)	0.005	27.1 (14.8, 52.3)	0.037
Non-SCC	51	32.8 (14.8, 51.7)		45.3 (24.2, 65.4)		20.8 (11.5, 42.1)	
**Stages**							
CIS+I	258	31.3 (14.2, 67.1)	<0.001	49.2 (24.2, 97.3)	<0.001	23.9 (14.0, 44.9)	0.002
IIA1	96	56.4 (28.4, 114.3)		80.9 (45.3, 124.4)		38.5 (14.2, 64.8)	
IIA2	27	51.1 (23.7, 87.5)		90.1 (41.4, 129.9)		35.8 (19.8, 49.7)	
IIB	18	55.0 (34.1, 78.9)		84.2 (65.4, 139.7)		28.7 (20.7, 45.3)	
III-IV	7	21.8 (7.1, 27.0)		62.0 (15.8, 68.7)		7.0 (5.4, 15.0)	
**Differentiation**							
High	144	24.5 (13.1, 59.2)	<0.010	42.7 (18.3, 93.3)	<0.010	20.4 (13.0, 38.7)	<0.010
Middle	131	46.1 (17.0, 82.0)		67.1 (32.6, 113.4)		27.2 (11.6, 49.7)	
Low	131	53.7 (23.8, 100.7)		79.2 (43.4, 118.4)		37.5 (16.8, 61.0)	
**Lymph gland**							
Negative	383	39.1 (15.5, 73.9)	0.003	54.8 (25.3, 100.7)	<0.001	25.3 (13.5, 47.9)	0.038
Positive	68	55.3 (23.7, 114.9)		100.7 (51.9, 139.7)		38.3 (15.5, 81.7)	
**Chemoradiotherapy**							
Non-CRT	208	31.5 (14.3, 73.9)	0.07	49.2 (21.1, 97.3)	<0.010	24.4 (14.4, 42.2)	0.27
CT or RT	82	50.0 (20.7, 84.8)		62.9 (37.5, 113.5)		31.3 (15.7, 53.7)	
CRT	116	47.8 (19.4, 83.0)		81.4 (45.5, 124.4)		29.5 (12.8, 58.7)	
**Age**							
≤57.5	271	40.5 (16.5, 72.8)	0.35	56.5 (25.6, 103.1)	0.23	25.1 (13.5, 48.7)	0.46
>57.5	135	45.2 (17.1, 87.0)		68.1 (30.6, 118.9)		27.1 (13.7, 52.1)	

**Table 2:** Description of univariate in clinicopathological features

Factor	N	CD20 median (IQR)	*P* value	CD57 median (IQR)	*P* value	CD68 median (IQR)	*P* value	CD163 median (IQR)	*P* value
**Pathological type**									
SCC	355	17.3 (7.3, 38.4)	0.1	14.3 (6.3, 34.6)	0.011	44.3 (21.3, 73.4)	0.46	59.5 (35.4, 92.9)	0.025
Non-SCC	51	14.3 (5.9, 21.2)		24.3 (10.9, 56.5)		35.5 (19.0, 60.8)		40.1 (28.0, 82.9)	
**Stages**									
CIS+I	258	17.4 (8.6, 37.4)	<0.001	19.8 (8.1, 44.5)	<0.001	35.8 (18.0, 63.2)	<0.001	50.1 (31.1, 82.5)	<0.001
IIA1	96	17.3 (6.1, 39.4)		13.0 (5.5, 26.1)		64.1 (30.6, 86.5)		75.6 (44.5, 118.7)	
IIA2	27	11.8 (4.9, 32.2)		10.1 (4.2, 15.3)		61.2 (45.2, 89.2)		79.6 (47.8, 100.4)	
IIB	18	12.6 (2.6, 26.9)		6.8 (3.4, 17.1)		62.0 (28.9, 96.8)		60.5 (34.4, 115.9)	
III-IV	7	2.6 (0.4, 4.1)		3.0 (1.9, 5.1)		28.6 (1.8, 62.6)		41.0 (37.4, 99.9)	
**Differentiation**									
High	144	16.7 (7.7, 43.1)	0.6	25.6 (9.3, 55.5)	<0.010	28.7 (15.7, 52.7)	<0.010	42.4 (28.7, 67.8)	<0.010
Middle	131	16.7 (6.9, 32.2)		12.4 (6.8, 24.9)		51.0 (27.1, 77.8)		62.9 (33.5, 97.5)	
Low	131	15.6 (6.5, 36.7)		12.3 (4.6, 28.6)		59.9 (26.2, 86.2)		74.1 (43.9, 111.3)	
**Lymph gland**									
Negative	338	16.8 (7.7, 37.4)	0.16	17.0 (7.0, 38.8)	0.014	39.7 (19.2, 67.6)	<0.001	52.5 (31.8, 88.2)	<0.001
Positive	68	12.7 (4.5, 34.4)		10.8 (4.9, 27.8)		67.7 (39.1, 89.2)		75.6 (42.7, 120.8)	
**Chemoradiotherapy**									
Non-CRT	208	17.4 (9.1, 43.1)	<0.010	22.7 (9.0, 52.1)	<0.010	33.2 (17.1, 57.2)	<0.010	49.2 (29.8, 76.2)	<0.010
CT or RT	82	14.7 (6.7, 28.2)		13.9 (5.2, 28.6)		48.8 (27.3, 82.2)		64.1 (38.3, 96.4)	
CRT	116	12.8 (4.5, 37.4)		9.7 (4.7, 19.5)		63.0 (32.7, 83.0)		76.4 (42.7, 109.5)	
**Age**									
≤57.5	271	16.9 (7.8, 39.8)	0.33	17.1 (6.8, 44.2)	0.01	41.5 (21.5, 68.1)	0.54	53.5 (33.1, 87.1)	0.13
>57.5	135	16.0 (6.1, 31.0)		13.2 (5.7, 25.2)		48.7 (19.1, 77.4)		65.5 (33.7, 104.7)	

**Table 3:** Cox regression analysis to estimate the risk score among different clinical variables

Variables	Univariate-Cox			Multiple-Cox		
	*P*	HR	95% CI	*P*	HR	95% CI
**Risk score**	0.001	5.470781	2.543-11.768	0.001	4.340013	1.894-9.943
**Pathological type**						
SCC		1.000				
Non-SCC	0.699	0.7898841	0.239-2.610			
**Stages**						
CIS+I		1.000			1.000	
IIA1	0.002	4.443537	1.722-11.464	0.213	1.933677	0.685-5.458
IIA2	0.004	6.049397	1.770-20.670	0.183	2.528847	0.646-9.895
IIB	0.000	11.36784	3.307-39.072	0.002	8.857496	2.166-36.227
III-IV	0.000	22.01399	5.676-85.386	0.001	25.23689	3.818-166.808
**Differentiation**						
High		1.000			1.000	
Middle	0.853	0.8937429	0.273-2.929	0.897	0.9056184	0.203-4.043
Low	0.008	3.458442	1.373-8.713	0.127	2.944453	0.736-11.788
**Lymph gland**						
Negative		1.000			1.000	
Positive	0.001	7.176987	3.448-14.939	0.027	2.764945	1.121-6.817
**Chemoradiotherapy**						
Non-CRT		1.000			1.000	
CT or RT	0.157	2.197865	0.739-6.540	0.815	0.8702612	0.271-2.796
CRT	0.001	4.50999	1.855-10.968	0.826	0.8863387	0.302-2.599
**Age**	0.013	2.527683	1.216-5.255	0.086	2.036392	0.905-4.582

**Table 4:** Low or high immune riskscore related with characteristics of patients in overall dataset

Factor	Low	High	*P* value
N	294	112	
**Status**			<0.001
live	284 (96.6%)	93 (83.0%)	
die	10 (3.4%)	19 (17.0%)	
**Pathological type**			0.096
SCC	252 (85.7%)	103 (92.0%)	
Non-SCC	42 (14.3%)	9 (8.0%)	
**Stages**			0.001
CIS+I	204 (69.4%)	54 (48.2%)	
IIA1	57 (19.4%)	39 (34.8%)	
IIA2	15 (5.1%)	12 (10.7%)	
IIB	13 (4.4%)	5 (4.5%)	
III-IV	5 (1.7%)	2 (1.8%)	
**Differentiation**			<0.001
High	125 (42.5%)	19 (17.0%)	
Middle	94 (32.0%)	37 (33.0%)	
Low	75 (25.5%)	56 (50.0%)	
**Lymph gland**			0.017
Negative	253 (86.1%)	85 (75.9%)	
Positive	41 (13.9%)	27 (24.1%)	
**Chemoradiotherapy**			<0.001
Non-CRT	167 (56.8%)	41 (36.6%)	
CT or RT	57 (19.4%)	25 (22.3%)	
CRT	70 (23.8%)	46 (41.1%)	
**Age**			0.045
≤57.5	205 (69.7%)	66 (58.9%)	
>57.5	89 (30.3%)	46 (41.1%)	

## Abbreviations

TC: tumor center
IM: tumor invasive margin
Th: T helper
CTL: cytotoxic T cell
NK: natural killer
RCC: renal cell carcinoma
TAMs: Tumor-associated macrophages
CT: chemotherapy
RT: radiotherapy
CRT: concurrent RT and CT
CIS: cervical carcinoma *in situ*
lasso: least absolute shrinkage and selection operator
SCC: squamous cell carcinoma
HR: hazard ratio
CI: confidence interval
HPV: human papillomavirus
