# Supplementary material for: Characteristics of Infiltrating Immune Cells and a Predictive Immune Model for Cervical Cancer
Source: J Cancer. 2021 Apr 20;12(12):3501–14. doi: 10.7150/jca.55970 (PMC8120169; doi:10.7150/jca.55970)
Supplement: Supplementary file 1 — Supplementary figures. [file jcav12p3501s1.pdf]

## Supplementary Data

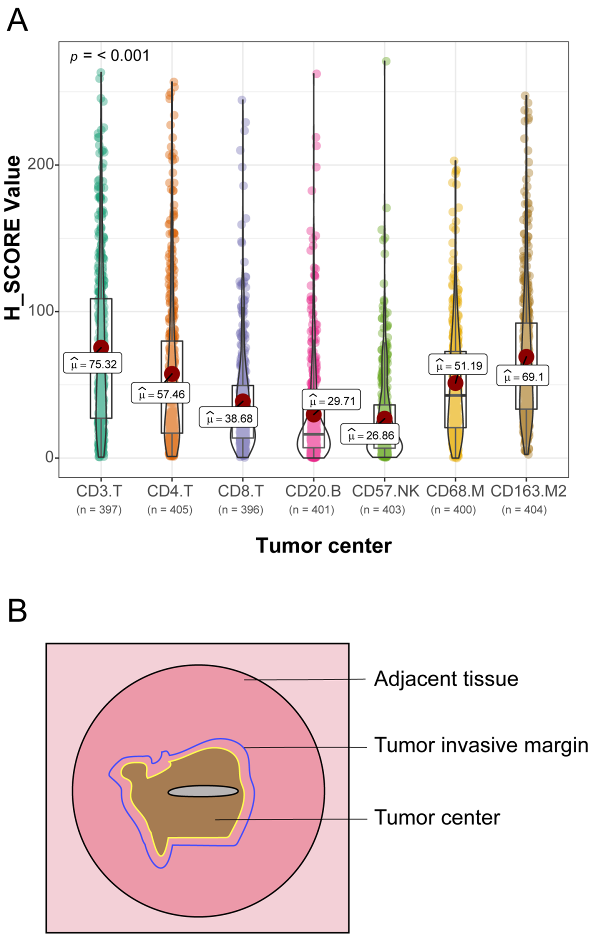


**Figure S1. H-SCORE of immune cells in the tumour centre and area chart** A, Expression levels of seven immune cell markers in the tumour centre. B, Illustration of the adjacent tissue and tumour centre.

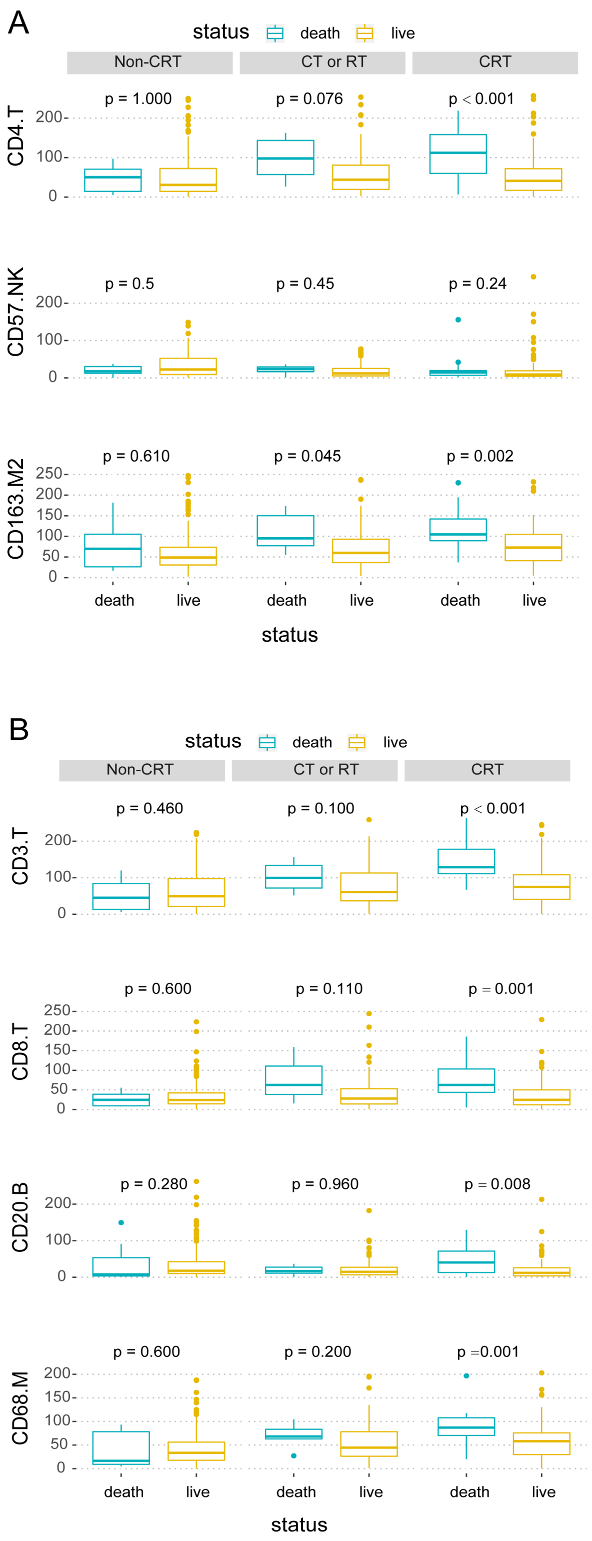


**Figure S2. Correlations between immune cells, chemoradiotherapy, and prognosis** A and B, Box chart displays comparison of the differences in infiltration of CD3+, CD4+, CD8+, CD20+, CD68+, and CD163+ cells in three treatment methods and patients with different prognosis.

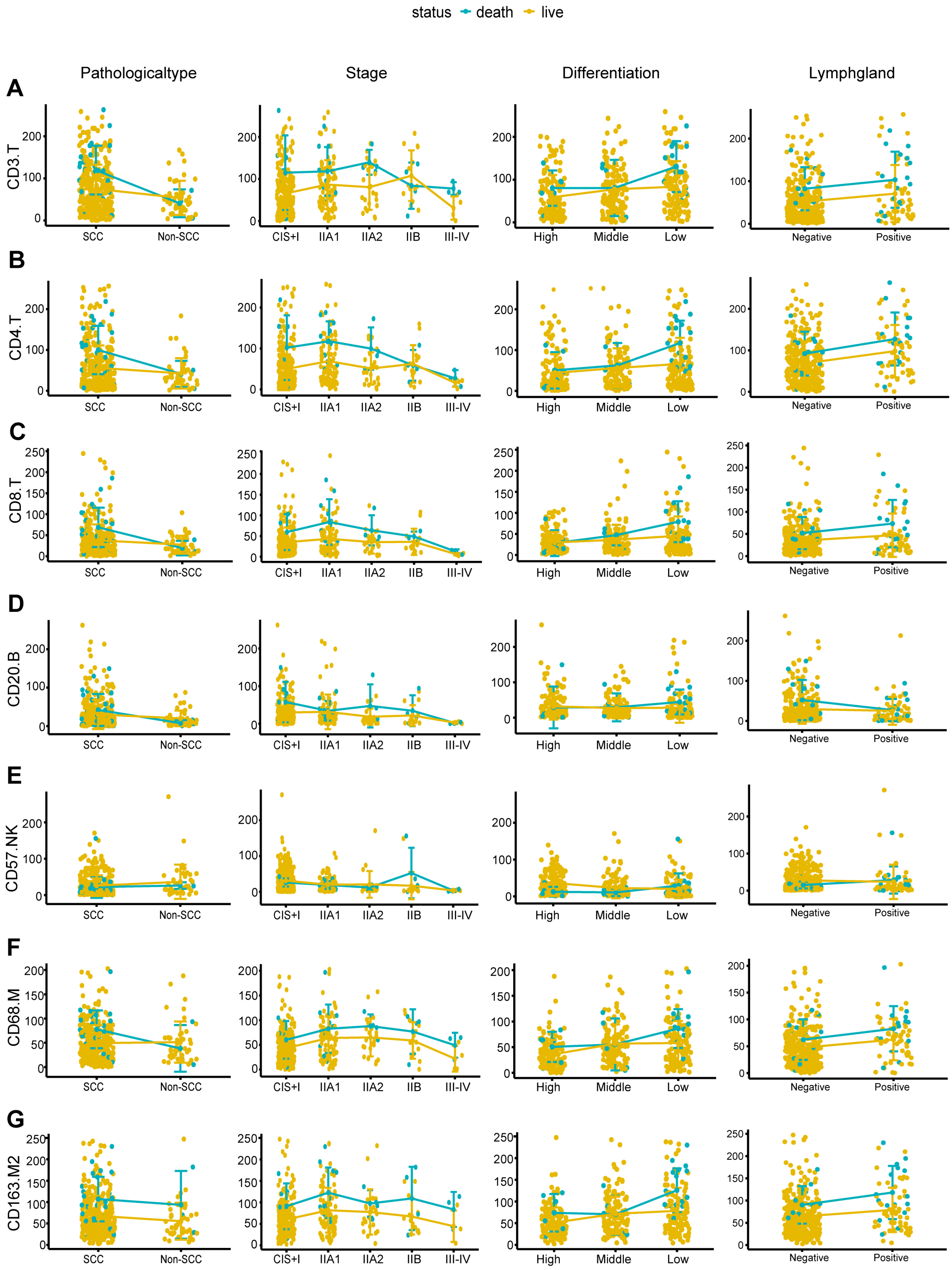


**Figure S3. Correlations between immune cell levels, clinicopathological characteristics, subtypes, and prognosis** Scatter chart showing the differences in clinicopathological characteristics between dead and living patients with respect to infiltration of CD3+, CD4+, and CD8+ T cells, CD20+ B cells, CD57+ NK cells, CD68+ macrophages, and CD163+ M2 macrophages.

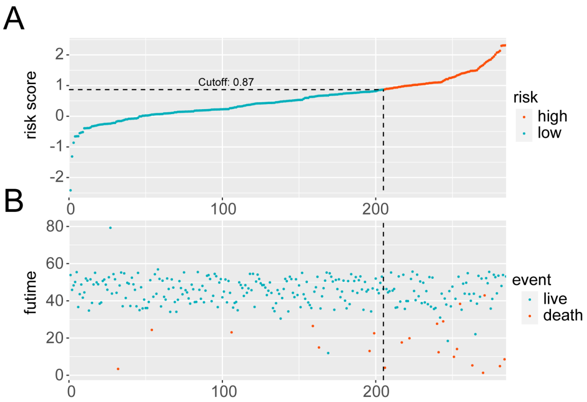


**Figure S4. The risk curve of the training queue.** A, The smooth curve shows the risk score threshold was 0·87 in the training queue. B, Survival after 36 months. Red and blue dots indicate deceased and living patients, respectively.

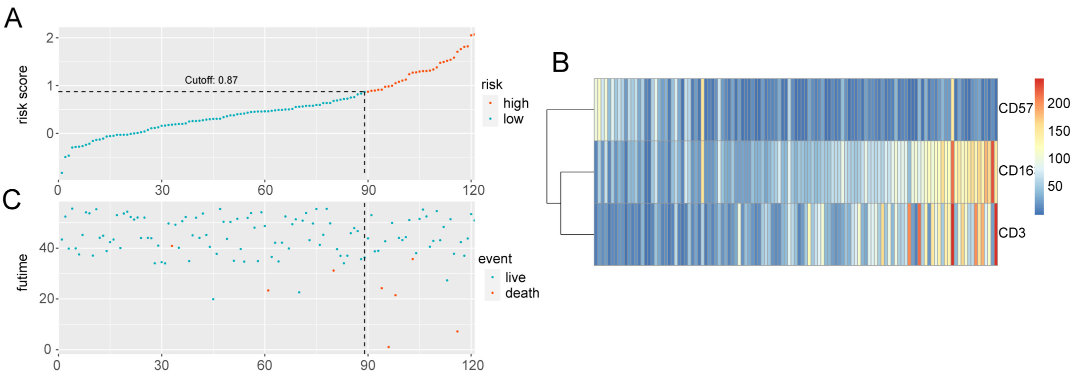


**Figure S5. Risk curve and heat map of the validation queue** A, The smooth curve indicates the risk score threshold (0·87) in the validation queue. B, Each point corresponds to a patient's survival time, with red and blue indicating deceased and living patients, respectively. C, Heat map of the levels of three immune cell types.

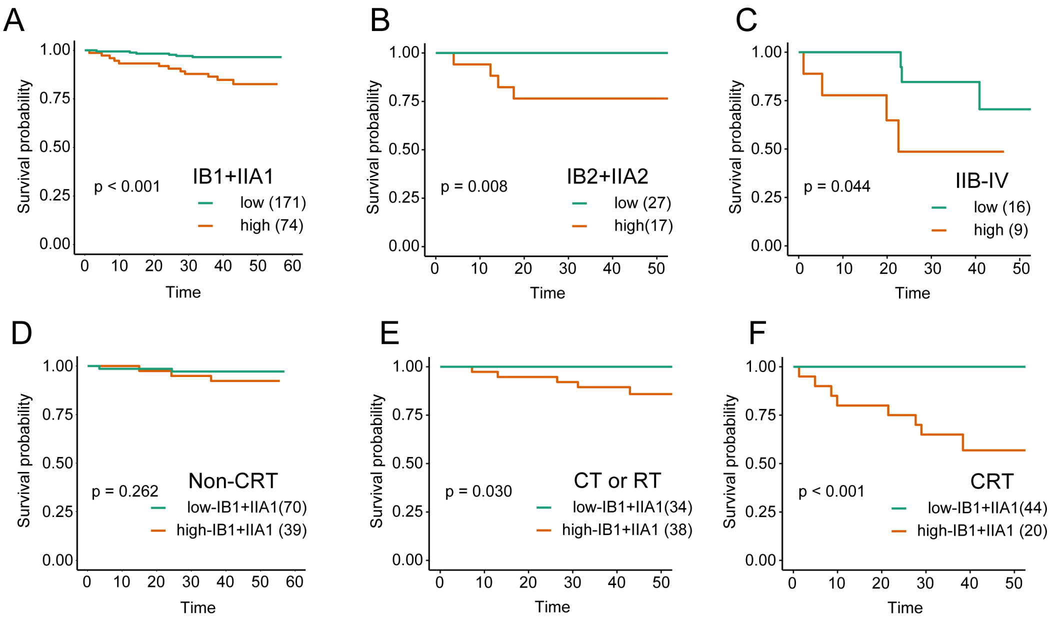


**Figure S6. Prognosis prediction in different stages and the effects of different treatments in stages IB1+IIA1** A, B, and C, Survival analysis showing the differences between patients with high risk scores and those with low risk scores in each of the three groups. E and F, Survival analysis showing the differences between patients with high risk scores and those with low risk scores in each of the IB1+IIA1 subgroups (i.e., non-CRT, CT or RT, and CRT).
